# Reviews

**Published:** 1881-11

**Authors:** 


### Reviews.

---

**Physicians' Visiting List for 1882.** Lindsay & Blackiston.

We have received a copy of this list which for thirty years has held a prominence over all others. The volume for 1882 contains the metric system of weights and measures, and a posological table, in addition to the usual contents.

**The Applied Anatomy of the Nervous System.** By AMBROSE L. RANNEY, A. M., M. D. With numerous illustrations. New York: D. Appleton & Co., 1, 3 and 5 Bond Street. 1881.

In the preface we learn that this volume comprises a course of lectures which were delivered by the author before the students of the medical department of the University of the City of New York, during the winter of 1880 and 1881. The volume abounds in illustrations and is in many respects a very interesting work. It is an admitted fact that the subject treated in this book is one sufficiently obscure to the profession generally, to make any work tending to elucidation most welcome. The treatise is a volume of 500 pages octavo. It is divided into four parts, treating respectively upon the brain, the cranial nerves, the spinal cord, and the spinal nerves. So far as we are able to judge, the work appears to notice all the latest researches in this department, and it is certainly one which the profession generally should possess. It has been written with a view to practical usefulness, and in this respect we are inclined to think that the efforts of the author have been successful. We earnestly recommend it as one unusually worthy of study.

---

**The Prescriber's Memoranda.** New York: Wm. Wood & Co.

The name of the compiler of this little work is not given on the title page. There is no preface, no introduction and no claim to have supplied "a want long felt." This unusual modesty caused us to look over the book pretty thoroughly, and we say that it is one of the most useful little books of this character, which has appeared. The writer, commencing with the first letter of the alphabet, gives Dr. Wm. T. Lusk's rules for the treatment of abortion, then some hints on the treatment of alcoholism, and so on, giving useful hints, memoranda and formulæ of approved prescriptions for various diseases, which are so arranged alphabetically as to be readily referred to.

